# The Associations between Yelp Online Reviews and Vape Shops Closing or Remaining Open One Year Later

**DOI:** 10.18332/tpc/67967

**Published:** 2017-02-02

**Authors:** Grace Kong, Jennifer Unger, Lourdes Baezconde-Garbanati, Steve Sussman

**Affiliations:** 1 Yale School of Medicine, New Haven, Connecticut, USA; 2 University of Southern California, Los Angeles, USA

**Keywords:** vape shops, Yelp, electronic cigarettes, retail, tobacco regulation

## Abstract

**INTRODUCTION:**

Vape shops are popular brick-and-mortar stores that sell e-cigarette products but are not understood well. Previous analysis of Yelp reviews of vape shops located in various ethnic neighborhoods in Los Angeles, California in 2014 identified characteristics of vape shop as delineated by consumers. In this study, we assessed the associations between these characteristics and vape shops going out of business in 2015.

**METHODS:**

Content analysis of Yelp reviews of 72 vape shops in 2014 identified 1) general characteristics of the reviews/reviewers, 2) vape shop, staff, and marketing attributes, 3) physical environment, and 4) health claims. In 2015, in-person visits confirmed that 22% of these vape shops closed permanently. We analyzed whether characteristics/attributes identified in 2014 associated with stores remaining open (n = 56) or permanently closing (n = 16) in 2015.

**RESULTS:**

Univariate findings showed that open vape shops relative to closed shops had greater 1) number of reviews, 2) rebuilds/fixings, 3) ratings of staff attributes as “helpful/patient/respectful,” and 4) report of the physical environment as “bar type.”

**CONCLUSIONS:**

Bar type vape shops and those with rebuilding/fixing capabilities were associated with staying open, suggesting the popularity of these attributes. Yelp consumer reviews is a useful research tool to identify consumer-determined important sustaining attributes of vape shops and may be used to identify aspects of enduring shops that need regulations.

## INTRODUCTION

E-cigarettes are battery-powered devices that vaporize a liquid solution (i.e., e-liquid) comprising propylene glycol (PG) and/or vegetable glycerin (VG), which may also contain nicotine and flavorings. While e-cigarettes have been sold by online retailers, in gas stations, in convenience stores, and in tobacco shops in the USA since 2007, a new type of brick-and-mortar shops referred to as vape shops started gaining popularity^1^. Vape shops are a fast growing and popular business. There are currently more than 10,000 vape shops in the USA^2^. According to market analysts, vape shops generated $900 million in revenue in 2014 and could be worth $10 billion by 2018^3^.

Vape shops not only sell e-cigarettes but they play a significant role in marketing e-cigarettes by creating a social milieu where employees/owners directly interact and communicate with local community residents/consumers to influence their perceptions about e-cigarettes and also to promote e-cigarette use^4^. Given that the field has not achieved consensus about risks and benefits of e-cigarettes, the role of vape shops on public health also remains unknown. Vape shops can pose potential harm to public health by encouraging e-cigarette use among non-users of tobacco or promote dual use among combustible tobacco users. Mishandling e-liquids and hardware by not taking safety precautions can also pose harm for employees and customers. Another concern is that vape shop employees tend to overestimate the safety and underestimate the harm of e-cigarettes^5, 6,^ and they are not adequately trained to access and interpret accurate health messages^4, 7^. On the other hand, if e-cigarettes are shown to be an effective harm reduction/cigarette cessation tool, vape shop owners and employees can help individuals to quit smoking to further decrease morbidity and mortality related to cigarette smoking. However, little is known about vape shop practices and more research is needed to understand how vape shops conduct their business.

Vapes hops have not been regulated until recently. Beginning August 8, 2016, the Food and Drug Administration (FDA) began regulating e-cigarettes^8^. The FDA can now prohibit the sale of e-cigarette to minors under the age of 18, ban free samples of e-liquids, and require manufacturers to obtain FDA authorization to market and distribute e-cigarettes and their related components (e.g., e-liquids, flavorings, cartridges, container of e-liquid, tank systems, drip tips, programmable software)^8^. These new regulations will influence vape shops inevitably. For instance, many vape shops may be unable to comply with the time-consuming and expensive process to obtain premarket approval if they “make, modify, modify, mix, manufacture, fabricate, assemble, process, label, repack, relabel, or import ENDS”^8^. Thus, many vape shops may begin to change other components of their business practices that do not require premarket approval to upkeep their business. Understanding consumer perceptions regarding important components of vape shops may shed light on how the vape shop industry may evolve to meet the regulatory demands^9^.

Consumer perceptions of vape shops can be assessed through the analysis of online Yelp reviews^10^. Yelp is a widely popular social networking website where users can locate and evaluate businesses by searching for a particular category within a geographic location. Users can read reviews of the products and services written by other users and also submit their own reviews. Yelp has 90 million unique visitors each month and more than 102 million reviews have been written^11^. Research studies have used Yelp to identify vape shops in major metropolitan cities in California^10, 12,^ New York^5^, Florida^13^, New Jersey^14^, and even across the USA^15^.

One of the first examination of Yelp reviews of vape shops in Los Angeles, California provided insight into vape shop practices and identified vape shop and staff attributes that consumers determined to be important^10^. Consumers found that important vape shop characteristics were a wide and unique selection of flavors/hardware, fair prices, and important staff characteristics were “friendly,” “helpful/patient/respectful,” and “knowledgeable/professional.” However, whether these consumer-determined characteristics actually impact the success or failure of the business is unknown. One means to assess the utility of such Yelp review characteristics would be to assess their associations with a practical outcome, such as vape shops going out of business over some period of time (e.g., one year later). In this study, we examined whether review characteristics (e.g., vape shop and staff characteristics) and other vape shop characteristics obtained outside of Yelp reviews in 2014 (e.g., neighborhood characteristics) were associated with whether vape shops remained in business or permanently closed in 2015.

In this study, Yelp review characteristics included average star ratings and vape shop characteristics (e.g., fair prices, device types, selection of flavors, venue type, amenities, marketing atmosphere) and staff attributes (e.g., “helpful,” “knowledgeable”) that Yelp reviewers discussed in their reviews. Given that adult e-cigarette users primarily use e-cigarettes to quit smoking or as an alternative to cigarettes^16^, we also included discussion on health claims (e.g., use of e-cigarette to quit smoking) on the reviews. We hypothesized that open shops, relative to closed shops in 2015, would have received higher star ratings, more comments indicating 1) a wide range of products (e.g., device type, flavors), 2) services available (e.g., ability to rebuild and fix devices), 3) positive staff attributes, 4) a more inviting physical environment, and 5) positive health claims in the reviews in 2014.

We also assessed factors obtained from other sources outside of Yelp. We assessed whether the neighborhood characteristics of the vape shops were associated vape shop outcomes. We hypothesized that vape shops in White neighborhoods would be more likely to remain open than those in other ethnic neighborhoods (e.g., African American, Latino, Korean) based on the findings that vape shops were more common in high-income neighborhoods with more White individuals^14, 17^, unlike traditional tobacco retailers that tended to be heavily located in low-income, ethnic minority neighborhoods ^e.g., 18^.

## METHODS

We searched Yelp for vape shops in diverse ethnic neighborhoods (i.e., Korean, African American, Latino, and White) in Los Angeles, California between March and June 2014. We used the US Census data to determine the racial/ethnic compositions of the neighborhoods in this city. The identified neighborhoods (e.g., Koreatown, Exposition Park, Commerce, Hermosa Beach) were then used as a filter on Yelp searches. The reviews were sorted from newest to oldest through the use of the “best match” filter.

**Figure 1:**
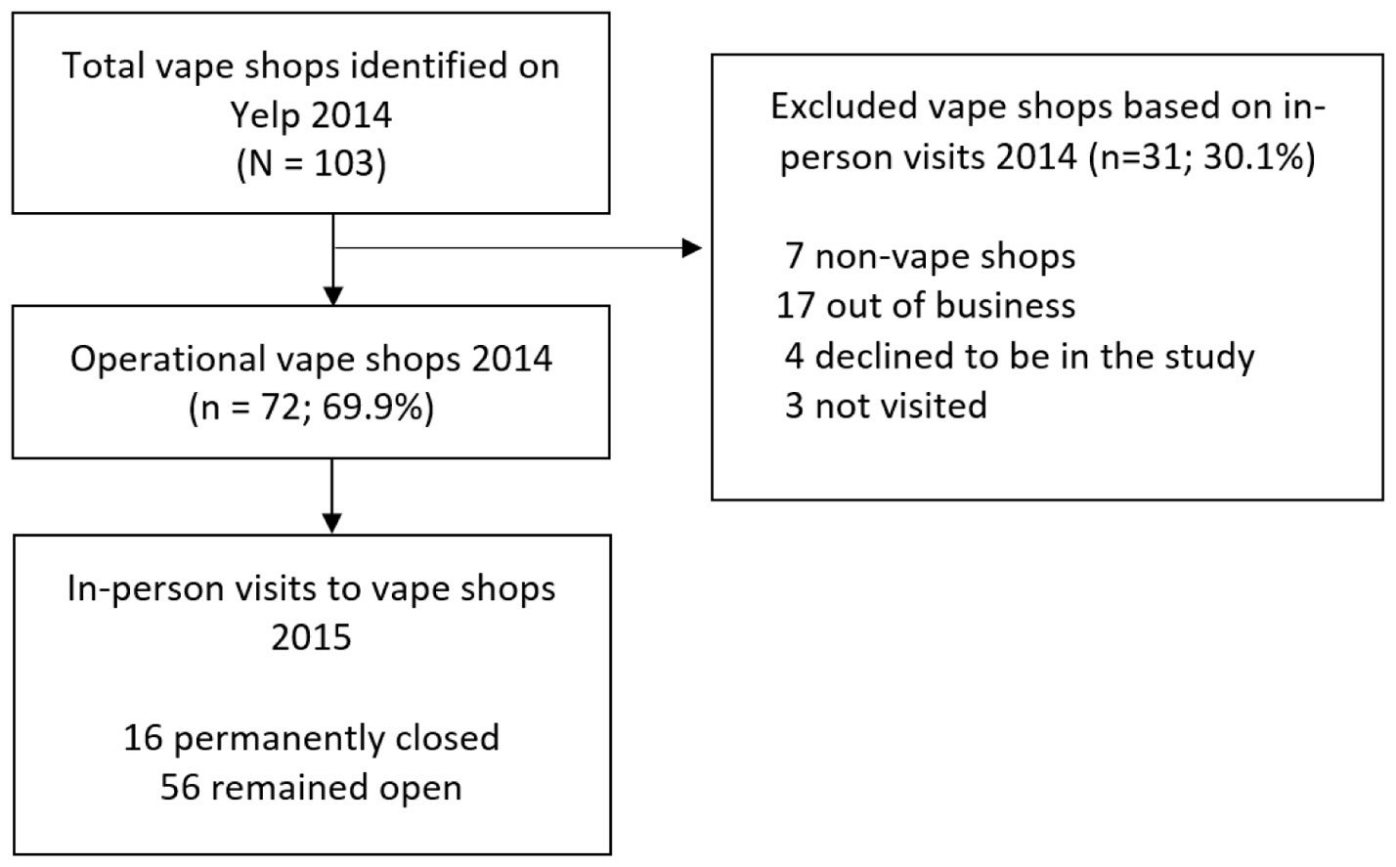
Reasons for exclusion of vape shops

The search yielded 103 vape shops with at least 5 reviews; 22 vape shops were in the most highly Korean neighborhoods in Los Angeles (ranging from 32% to 8% Korean), 30 vape shops were in the most highly African American neighborhoods (ranging from 38% to 14% African American), 25 vape shops were in the most highly Latino neighborhoods (ranging from 93% to 63% Latino), and 26 vape shops were in the mostly highly White neighborhoods (ranging from 85% to 70% White). In-person visits to these vape shops identified that 31 were non-operational (See Figure 1 for specific reasons). In 2015, exactly one year after the previous search, in-person visits to 72 of these vape shops identified that 16 vape shops permanently closed. For the current analysis, up to first 20 Yelp reviews of 72 vape shops in 2014 were analyzed. The average number of reviews analyzed in this study was 16.47 (SD=4.47). See^10^ for further information on the description of Yelp review data extraction methods and the development of coding measures.

### Coding Measures

Study variables were open-ended (see^10^ for detailed information on coding measures and intercoder reliability). In sum, Cohen’s Kappa ranged from 0.21–0.81 and we excluded characteristics that had Kappas lower than 0.30 (e.g., other characteristics, quick service, other physical environment attributes, bad furniture). Below lists a summary of the coding measures examined in this study.

Reviewer information included reviewer’s sex based on the name and the picture provided on his/her Yelp profile.

General review information included the total number of reviews provided per vape shop and star ratings (number of 5 stars, 4 stars, 3 stars, 2 stars, 1 star).

Characteristics noted as important in vape shop included never being rushed by employees, a wide range of nicotine concentration, fair prices, ability to rebuild/fix devices (i.e., usually involves altering purchased devices to permit better vapor flow), on-line store capability, a wide selection of flavors (juices) or hardware, and unique flavors or hardware.

Staff attributes included characteristics such as “helpful/patient/respectful,” “knowledgeable/professional,” “friendly,” good personality, quick service, “lets customers try flavors,” and other.

Physical environment included three broad categories: 1) venue type, such as bar, club, and other, 2) venue amenities, such as parking, cleanliness, furniture, lighting, art, music, TVs, water tank, mugs, chalkboard menu, and other, and 3) marketing atmosphere, such as chic/classy, relaxed, fun, “awesome (stated by the reviewer),” and other.

Health claims included claims that e-cigarettes are relatively safe and one can quit smoking using an e-cigarette.

### Data analysis

Using SPSS version 21, we conducted descriptive statistics on study variables and chi-square tests (for categorical variables) and t-tests (for continuous variables) to assess the differences in characteristics identified in 2014 between vape shops that remained open vs. closed in 2015. We dichotomized responses if a characteristic was endorsed fewer than two times (1=endorsement of the characteristic, 0=no endorsement of the characteristic). We then conducted bivariate correlation analyses among variables that were significantly associated with the outcome in chi-square and t-tests.

## RESULTS

See Table 1 for the percentages/means and standard deviations and chi-square/t-test statistics comparing open shops vs. closed shops on review/reviewer attributes, neighborhood characteristics, vape shop characteristics, staff attributes, physical environment, and health claims. Open vape shops had more total reviews (assessed in 2014) than vape shops that closed in 2015 (p=0.001). Average star ratings for all shops were 3.06 (SD=0.85), and 57.4% (SD=13.9%) of the reviewers were men, 26.9% (SD=13.4%) were women, and 15.7% (SD=0.9%) were “unknown.” Approximately, 29.2% of vape shops were in African American, 22.2% in Latino, 18.1% in Korean, and 30.6% in White neighborhoods. Open and closed shops did not differ in the average star ratings (p=0.36), in the proportion of male/female reviewers, (p=0.45), and in the neighborhood characteristics (p=0.501).

Three notable differences were observed: open shops relative to closed shops had 1) greater endorsement of the ability to rebuild and fix devices, 2) more mention of staff attributes as “helpful/patient/respectful,” and 3) greater report of the physical environment as a “bar type.” Correlation among variables showed that the total number of reviews was associated with staff attributes of “helpful/patient/respectful” (r(72)=0.45, p=0.001) and physical environment as a “bar type” (r(72)=0.28, p=0.019), and “bar type” was associated with “helpful/patient/respectful” (r(72)=0.29, p=0.012). We did not observe other significant associations among variables.

## DISCUSSION

Vape shop and staff attributes identified through Yelp reviews in 2014 were associated with vape shop outcomes one year later. For instance, vape shops with low number of Yelp reviews were more likely to permanently close one year later. Low number of reviews could represent low popularity. Contrary to our hypothesis, we did not observe that vape shops located in White neighborhoods were more likely to remain open than those located in other ethnic neighborhoods (i.e., African American, Latino, Korean). Although previous literature has shown that vape shops are less likely to be located in Latino and African American neighborhoods^17^, our study findings indicate that the business outcome does not appear to be associated with ethnic/racial compositions of the neighborhoods. However, it is important to note that since vape shops included in this study were in Los Angeles, California, the findings may not generalize to other geographic regions. Future research should examine neighborhood characteristics of vape shops in other geographic regions.

We observed that vape shops that remained open were more likely to be described as a “bar type” than shops that closed. Perhaps, an appealing component of vape shops is not only the types of devices and e-liquids that are being sold but also the ambiance of the physical environment that vape shops create for users^1^. Vape shop patrons may be using vape shops as a space to spend time with friends and socialize by vaping together. The finding that vape shops that resemble bars are more likely to stay open, suggests that this type of layout is an important component to users. This is not surprising, perhaps, given that hookah bars, type of bars that combine alcohol and hookah tobacco, are popular among US young adults^19^. More research is needed to explore aspects of “bar type” that appeals to vape shop patrons. We also observed that “bar type” was associated with staff characteristics of “helpful/patient/respectful,” which suggests that employees of these shops have a positive interaction with their customers. However, décor and service characteristics may be also important for the success of other type of businesses and may not be solely related to the success of vape shops. Studies using Yelp to predict success of other type of businesses, such as restaurants, have shown similar attractive characteristics^20,21^. Specifically, evaluation of Yelp reviews revealed that service and the décor of restaurants were important features. In addition, features unique to restaurants, such as the ability to take out were also identified^21^.

Similarly, we also identified important features specific to vape shops. Relative to closed shops had more mention of the ability to rebuild or fix devices in the reviews identified one year prior to closing. The appeal of the ability of vape shops to rebuild and fix devices indicates the popularity of consumers using vape shops for these purposes. However, the Food and Drug Administration (FDA)’s new rules, which requires any vape shops that mix e-liquids, make or modify any type of devices to obtain approval from the FDA to sell their products^8^, will inevitably affect vape shop practices given that vape shops often customize e-cigarette flavorings and devices. While these rules went into effect August 8, 2016, the manufacturers will have two years to submit the application materials to obtain approval. During this grace period, vape shops may need to start to adapt their business practices. Many small vape shops may be unable to comply with this cumbersome and expensive process so they may rely on other methods such as improving ambiance that may appeal to users more and using other marketing strategies to attract consumers. For instance, vape shops are known to host events such as cloud chasing contests, which draw vape enthusiasts^12^. However, other marketing strategies to attract consumers are relatively unknown. Close monitoring is needed to detect changes in vape shop marketing strategies and business practices to inform regulation.

Study limitations should be taken into account when interpreting the findings of this study. First, Yelp does not have information on when the vape shops first opened so we could not address the effect of time. Vape shop open date could be related to the number of total Yelp reviews received, as well as whether vape shops remained open or closed in 2015. However, the first concern is mitigated by our examination of the first 20 most recent reviews. Second, although Yelp reviews are useful in identifying consumer perceptions regarding vape shops^10^, there likely are other factors not measured in Yelp reviews that influenced the shops to remain open or closed. For instance, the built environment, general location, and poor business practices could contribute to going out of business.

Third, review frauds (i.e., creating overly positive reviews for themselves or bad reviews for their competitors) do occur^22^. However, Yelp attempts to remove fake reviews by using an automatic algorithm to flag suspicious reviews and filter them from appearing on the main Yelp website^22^. Fourth, although our experience with vape shops indicate that the majority of vape shops are individually owned, some could be part of a multiple-store organization so if a multiple-store vape shop went out of business then the closure of vape shops part of this organization could be counted more than once.

Despite these limitations, the strength of this study is that consumer perceptions identified in Yelp reviews were associated businesses remaining open or going out of business one year later. Yelp may be a useful tool to assess evolving vape shop practices and consumer perceptions. Examination of vape shop practices is important because vape shops not only sell e-cigarette products but they communicate/market appealing features of the product to consumers and local community, create positive social norms surrounding e-cigarettes, and have the ability to disseminate health information. Given the likely changes in vape shop practices with the new FDA regulations, future studies should examine the changing trends in vape shop sales and marketing practices. The marketing and communication strategies used by vape shops may also shed light on e-cigarette prevention strategies.

## Figures and Tables

**Table 1:** 2014 Yelp review ratings of characteristics of vape shops of those that closed versus that remained open in 2015.

	Open (n=56)	Closed (n=16)	Chi-square/t-test, p-value
**Review/Reviewer attributes**			

Total reviews (M, SD)	42.43, 49.74	18.69, 9.26	3.73, p = 0.001
Star ratings (M, SD)	3.11, 0.84	2.89, 0.89	0.92, p = 0.36
Proportion of male/female sex (M, SD)	0.57, 0.13	0.60, 0.15	−0.76, p=0.45
Vape shop neighborhood characteristics			2.36, p=0.501
White	26.8%	43.8%	
African	32.1%	18.8%	
American			
Latino	21.4%	25.0%	
Korean	19.6%	12.5%	

**Characteristics important in this vape shop**			

Great flavor or hardware selection M, SD)	6.54, 2.62	6.31, 3.32	0.28, p = 0.778
Fair prices (M, SD)	4.00, 2.29	3.81, 2.69	0.29, p = 0.782
Unique flavors or hardware (M, SD)	3.66, 2.31	3.00, 2.22	1.02, p = 0.313
Rebuilds or fixing (M, SD)	1.96, 1.56	1.13, 1.15	2.00, p = 0.05
Never rushed (%)	26.8%	12.5%	1.41, p = 0.235
On-line store capability (%)	10.7%	6.2%	0.28, p = 0.595
Wide range nicotine (%)	3.6%	6.2%	0.22, p = 0.636

**Staff attributes mentioned**			

Friendly (M, SD)	4.71, 2.01	5.63, 2.80	−1.21, p = 0.239
Helpful/patient/respectful (M, SD)	7.61, 3.46	5.69, 2.98	2.02, p = 0.048
Knowledgeable/professional (M, SD)	5.27, 2.71	4.88, 1.82	0.68, p = 0.504
Good personality (e.g., cool, relaxed) (M, SD)	1.66, 1.20	1.31, 1.40	0.99, p = 0.326
Let me try out lots of flavors (M, SD)	2.48, 1.89	2.44, 1.82	0.84, p = 0.933
Other attributes (M, SD)	7.45, 3.12	6.31, 3.11	1.28, p = 0.20

**Physical environment and amenities**			

Clean (M, SD)	.82, .84	.75, .86	0.30, p = 0.765
Bar type (M, SD)	1.14, 1.63	.25, .77	3.07, p=.003
Club type (%)	1.8%	0.0%	0.29, p = 0.590
Good parking (%)	39.3%	43.8%	0.10, p = 0.748
TVs (%)	23.2%	31.2%	0.43, p = 0.525
Art (%)	17.9%	37.5%	2.79, p = 0.096
Lighting (%)	19.6%	6.2%	1.61, p = 0.205
Other venue type (M, SD)	.89, 1.40	1.19, 1.91	−0.68, p = 0.496

**Atmosphere**			

Relaxed (M, SD)	1.05, 1.10	1.56, 1.15	−1.61, p = 0.111
Awesome (%)	12.5%	12.5%	0.00, p = 01.00
Chic/classy (%)	7.1%	6.2%	.015, p = 0.901
Fun (%)	8.9%	0.0%	1.54, p = 0.215
Other (M, SD)	1.82, 1.56	1.94, 1.61	−0.26, p = 0.795

**Health claims**			

Can quit smoking here (%)	39.3%	31.2%	0.34, p = 0558
E-cigarettes are safe (%)	17.9%	12.5%	0.26, p = 0.612
